# 3DPAFIPN as a halogenated dicyanobenzene-based photosensitizer catalyzed gram-scale photosynthesis of pyrano[2,3*-d*]pyrimidine scaffolds

**DOI:** 10.1038/s41598-023-40360-w

**Published:** 2023-08-12

**Authors:** Farzaneh Mohamadpour

**Affiliations:** School of Engineering, Apadana Institute of Higher Education, Shiraz, Iran

**Keywords:** Photocatalysis, Chemistry, Organic chemistry

## Abstract

Utilizing the Knoevenagel–Michael tandem cyclocondensation reaction of barbituric acid/1,3-dimethylbarbituric acid, malononitrile, and aryl aldehydes, a sustainable methodology for the photosynthesis of pyrano[2,3-*d*]pyrimidine scaffolds has been devised. The present study expounds on the development of a green radical synthetic approach toward this class of compounds. In this study, a novel halogenated dicyanobenzene-based photosensitizer was utilized in an aqueous solution, exposed to air at room temperature, and activated by a blue LED as a renewable energy source for the purpose of generating energy. The primary aim of this endeavor is to employ a recently developed, easily obtainable, and affordably priced halogenated cyanoarene-based donor–acceptor (D–A). The 3DPAFIPN [2,4,6-tris(diphenylamino)-5-fluoroisophthalonitrile]} photocatalyst, as a thermally activated delayed fluorescence (TADF), is capable of inducing single electron transfer (SET) upon irradiation with visible light, thereby offering a facile and efficient approach with a high degree of effectiveness, energy efficiency, and eco-friendliness. The aforementioned phenomenon facilitates the exploration of the temporal changes that have occurred in the interactions between the surroundings and chemical constituents. The present study aimed to investigate the turnover number (TON) and turnover frequency (TOF) for pyrano[2,3-*d*]pyrimidine scaffolds. Additionally, it has been demonstrated that gram-scale cyclization is a viable method for utilization in industrial applications.

## Introduction

In contemporary literature, photoredox catalysis has been leveraged as an origin of pioneering methodologies within the domain of organic chemistry^1–4^. The field of photoredox catalysis, which involves the integration of metal-promoted reactions with photoredox cycles, has garnered considerable attention from both academia and industry^5^. The strategic topic of research involves the utilization of inexpensive, readily synthesized, and efficient organic dyes to facilitate the development of novel, effective, and selective metal-promoted reactions^6^. Within this domain, the organic dyes must strive to supplant the extensively utilized inorganic complexes reliant upon Ir(III) and Ru(II). These complexes are notable for their protracted excited state durations, which may incline toward dynamic quenching when juxtaposed with organic molecules. Typically, organic dyes exhibit shorter excited state lifetimes, a significant impediment in the formulation of effective photoredox mechanisms. The scientific community has exhibited a significant interest in a distinct group of organic chromophores owing to their notable characteristics and effectiveness^7^. The molecules under consideration exhibit a unique characteristic known as thermally activated delayed fluorescence (TADF), which is observed in molecules that have a negligible energy gap (typically less than 0.2 eV) between their lowest two excited states, i.e., S_1_ and T_1_. At ambient conditions, the phenomenon of reverse intersystem crossing (RISC) from the triplet excited state (T_1_) to the singlet excited state (S_1_) occurs in the molecules under consideration, facilitated by a thermally activated pathway. This results in the production of a delayed fluorescence phenomenon, which is commonly observed in such systems. The task at hand concerns the coupling of the notable efficiency of reduced instruction set computing (RISC) with the commendable quantum yield of fluorescence. The year 2012 saw a significant contribution to the field of organic light-emitting diodes (OLEDs) through the publication of a seminal paper by Adachi^8^. This paper reports on the successful development of dicyanobenzene molecules with desirable photophysical properties, and their demonstrated applications in OLEDs. Subsequent to these initial findings, analogous TADF chromophores have been implemented across diverse domains, such as photocatalysis^7,9^. Owing to the facile manipulability of their redox potentials and the protracted singlet excited states arising from TADF, isophthalonitriles represent a promising class of chromophores for deployment as organic photocatalysts, facilitating numerous chemical transformations^10^. The compound 2,4,6-tris(diphenylamino)-5-fluoroisophthalonitrile (3DPAFIPN) has been increasingly utilized in various synthetic procedures that are activated by visible light. Examples of such protocols include intramolecular cyclizations^11,12^ as well as the formation of C–C^13,14^, N–C^15^, and P–C^16^ bonds^5^.

Owing to its copious energy reserves, economical expense, and the potential to access sustainable energy sources, visible light irradiation is deemed to be a reliable method for synthesizing organic compounds^17–19^.

It is expected that pyranopyrimidines will demonstrate compelling pharmacological and biochemical characteristics such as an inhibitor of the antiallergic^20^, antihypertensive^21^, cardiotonic^22^, bronchiodilator^23^, antibronchitic^24^, and antitumor activities^25^.

Numerous catalysts exhibit the capability to generate artificial pyrano[2,3-*d*]pyrimidine frameworks, such as DABCO-based ionic liquids^26^, L-proline^27^, iron ore pellet^28^, nano-sawdust-OSO_3_H^29^, Al-HMS-20^30^, TSA/B(OH)_3_^31^, Mn/ZrO_2_^32^, cellulose-based nanocomposite^33^, DBA^34^, TBAB^35^, Fe_3_O_4_@SiO_2_@(CH_2_)_3_-Urea-SO_3_H/HCl^36^, Et_3_N-Ultrasonic^37^, ZnFe_2_O_4_ nanoparticles^38^, microwave^39^, nickel nanoparticles^40^, CaHPO_4_^41^, Zn[(L)proline]_2_^42^, theophylline^43^, β-CD^44^, and CuO/ZnO nanocatalyst^45^. Various factors, such as protracted reaction times, the employment of costly reagents, intricate reactions, and diminished yields, exert significant impacts on waste handling and disposal practices. Moreover, the task of isolating homogeneous catalysts from reaction mixtures can present a formidable challenge. Today, the application of visible-light and photoreactions has attracted the attention of researchers^46–57^. The present study describes the utilization of photocatalysts in the production of heterocyclic compounds, emphasizing the implementation of environmentally sustainable practices. Based on the investigation, halogenated organic dye photo-redox catalysts are also readily attainable and economically feasible. The aforementioned technique results in the utilization of a robust donor–acceptor (D–A) cyanoarene as a potent organo-photocatalyst.

The primary research focus was on 2,4,6-tris(diphenylamino)-5-fluoroisophthalonitrile (3DPAFIPN), owing to its exceptional photophysical and photochemical properties. The advent of dicyanobenzene-based photosensitizers as a thermally activated delayed fluorescence (TADF) and possessing remarkable photoelectric behavior, has expanded the range of available photocatalysts for organic chemists.

The current study has identified a novel halogenated cyanoarene-based photosensitizer, namely 3DPAFIPN, as a donor–acceptor (D–A) photocatalyst which operates via a consecutive visible-light-induced electron transfer process. The protocol herein utilizes the domino Knoevenagel–Michael three cyclocondensation reaction involving barbituric acid/1,3-dimethylbarbituric, malononitrile, and aryl aldehydes. Furthermore, this reaction can exploit blue LED as an eco-friendly and renewable energy source in an aqueous medium. Despite the smooth and timely completion of all tasks and adherence to the approved financial plan.

## Experimental

### General

The melting points of the various compounds were determined using a 9100 electrothermal apparatus. The Bruker DRX-400 and DRX-300 Avance instruments were employed for the acquisition of ^1^HNMR spectra utilizing DMSO-d_6_. The aforementioned substances were graciously provided in substantial quantities by Fluka, Merck, and Acros, and were instantaneously employed.

### This study presents a methodology for the green production of pyrano[2,3-d]pyrimidines (4a-f)

At ambient temperature, a solution comprising 3 mL of water and 0.2 mol% of 3DPAFIPN was prepared. The mixture was subsequently combined with barbituric acid/1,3-dimethylbarbituric acid (**3**, 1.0 mmol), malononitrile (**2**, 1.0 mmol), and aryl aldehydes (**1**, 1.0 mmol). The responses were documented by means of thin-layer chromatography (TLC). Subsequent to the chemical reaction, the unrefined solid was subjected to screening, and subsequent washing with water, followed by crystallization from ethanol, thereby obviating the requirement for supplementary purification techniques. The present inquiry pertains to the feasibility of producing the aforementioned compounds on the gram-scale through the avenue of pharmaceutical process research and development (R&D). In a single experiment, a combination of 3-nitrobenzaldehyde, malononitrile, and barbituric acid at a molar amount of 50 mmol was utilized. Following a reaction period of 3 min, the resultant product was retrieved through the implementation of a conventional filtration method. Based on the ^1^HNMR spectroscopy data, the chemical compound in question exhibits a high degree of spectroscopic purity. The Supporting Information file provides a spectroscopy file.

## Results and discussion

**Figure 1:**
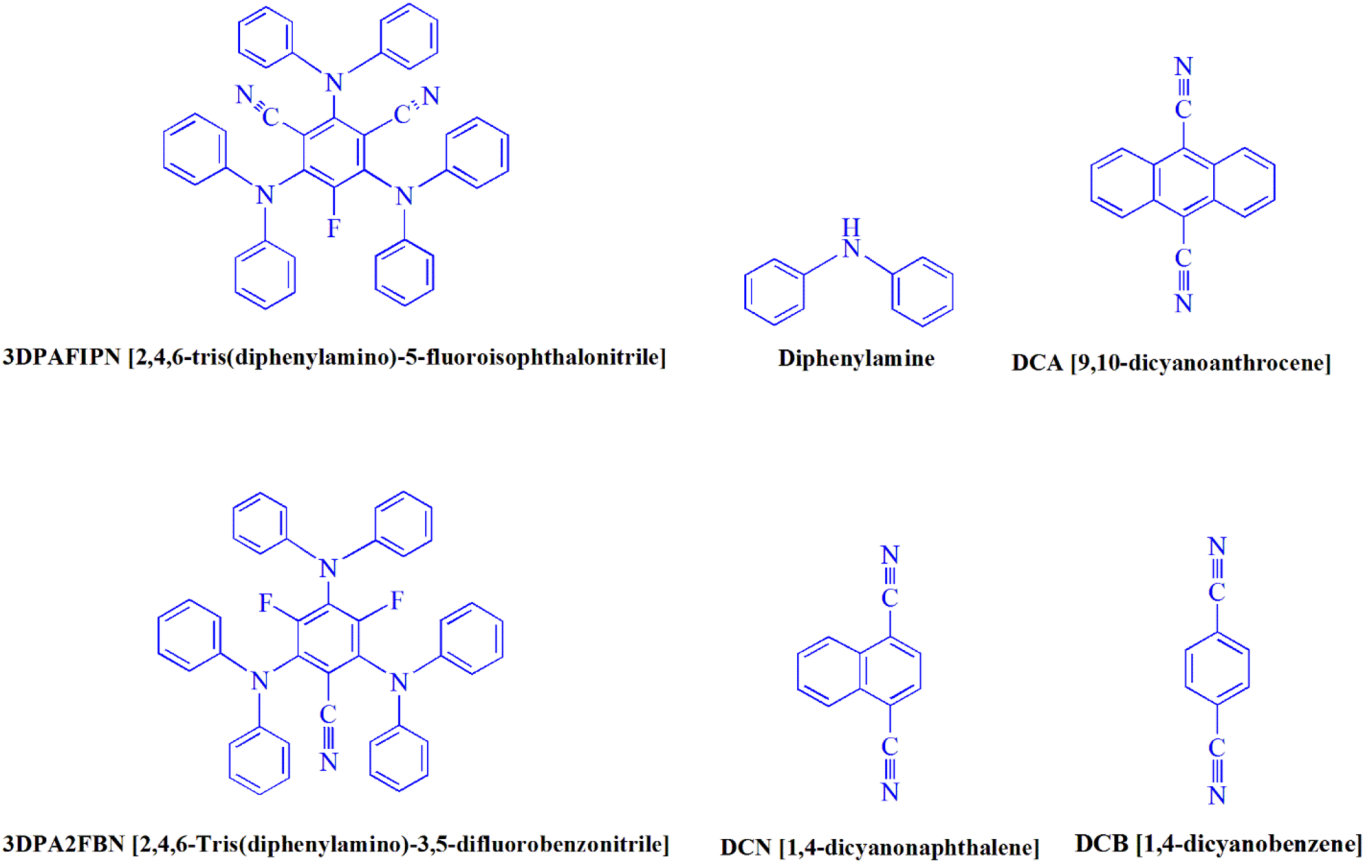
In this study, the catalyst's adequacy was surveyed.

**Figure 2:**
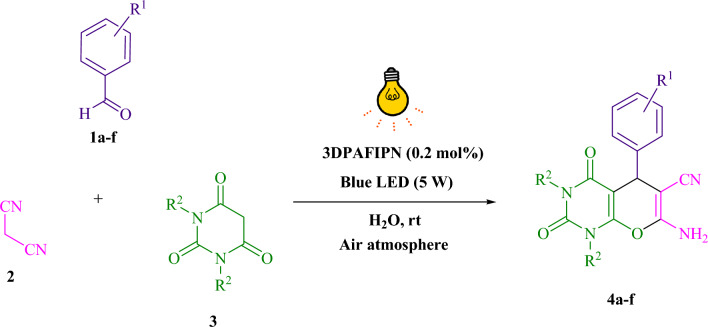
A methodology for the radical synthesis of pyrano[2,3-*d*]pyrimidine scaffolds is herein presented.

The present study investigated the reaction of benzaldehyde, malononitrile, and barbituric acid within a 3 mL aqueous medium. Through the process of incubation of 3 mL of water without the involvement of a photocatalyst for a period of 15 min, a quantity of **4b** amounting to 21% was produced at room temperature. A detailed account of this observation is presented in Table 1, entry 2. The incorporation of several supplementary photocatalysts facilitated the reaction rate. The data illustrated in Fig. 1 exhibits the constituting substances to be 3DPAFIPN, diphenylamine, DCA, 3DPA2FBN, DCN, and DCB. The present methodology allows for the production of **4b** with varying yields. The aforementioned results facilitated a higher level of operational efficiency for 3DPAFIPN. As per the data provided in Table 1, entry 1, a reaction comprising 0.2 mol% of 3DPAFIPN resulted in a 97% yield. Table 2 displays discernibly inferior outcomes for MeOH, DCM, EtOAc, CH_3_CN, DMSO, THF, EtOH, toluene, and solvent-free conditions exhibited a notable enhancement in the productivity and expeditiously facilitated the procedure. In the context of H_2_O, the reaction was observed to exhibit a notably high rate and consequential yield. Based on the statistical data presented in Table 2, specifically entry 1, a yield of 97% was achieved. Various light sources have been employed in studies aimed at investigating the impact of blue light on crop yield, as documented in Table 2. During the assessment that was carried out without the implementation of an illuminating apparatus, the presence of **4b** was detected in the diminutive quantity. The present investigation demonstrates that the co-presence of 3DPAFIPN and visible light is an imperative prerequisite for the successful synthesis of product **4b**. In order to determine the optimum configurations, levels of blue light-emitting diode (LED) intensities at 3 W, 5 W, and 7 W were employed. Based on the findings of the study, it was determined that the implementation of blue light-emitting diodes (LEDs) with a power output of 5 watts yielded the most favorable results. Experiments were performed on several substrates under idealized conditions, depicted in Table 3 and Fig. 2. The addition of a benzaldehyde substituent did not have a significant impact on the resultant reaction outcome. In the present reaction, the substitution of halide functionality was deemed permissible. The current state of the reaction permits both reactions that involve functional groups capable of electron donation and those that involve functional groups that exhibit electron withdrawal. The potential yield of *ortho*-, *meta*-, and *para*-substituted aromatic aldehydes is remarkably elevated in nature. The reactivity observed in both barbituric acid and 1,3-dimethylbarbituric acid was found to be identical. Table 4 presents definitive value measures of the turnover frequency (TOF) and turnover number (TON). The two distinct types of yield, namely, Yield/Amount of catalyst (mol), Yield/Time/Amount of catalyst (mol), are often expressed in the form of TON and TOF, respectively, in academic writing. Increased values of turnover number (TON) and turnover frequency (TOF) have the capacity to enhance catalyst performance, as they reduce the amount of catalyst required to promote desirable yields. Regarding **4b**, a TOF of 161.6 is considered high while a TON of 485 is regarded as high as well. The objective of the investigation was to optimize productivity and minimize reaction durations while mitigating catalyst usage to the lowest extent feasible.

**Table 1:** A photocatalyst optimization table is provided herein for the production of **4b**^a^.


Entry	Photocatalyst	Solvent (3 mL)	Time (min)	Isolated yields (%)
1	3DPAFIPN (0.2 mol%)	H_2_O	3	97
2	–	H_2_O	15	21
3	Diphenylamine (0.2 mol%)	H_2_O	3	34
4	DCA (0.2 mol%)	H_2_O	3	29
5	3DPA2FBN (0.2 mol%)	H_2_O	3	83
6	DCN (0.2 mol%)	H_2_O	3	25
7	DCB (0.2 mol%)	H_2_O	3	23
8	3DPAFIPN (0.1 mol%)	H_2_O	3	82
9	3DPAFIPN (0.3 mol%)	H_2_O	3	97

^a^Reaction conditions: several photocatalysts were combined with a quantity of one millimole each of barbituric acid, benzaldehyde, and malononitrile at room temperature.

**Table 2:** The optimization table of solvent and visible light conditions for the synthesis of **4b**^a^.


Entry	Light source	Solvent (3 mL)	Time (min)	Isolated yields (%)
1	Blue light (5 W)	H_2_O	3	97
2	–	H_2_O	10	Trace
3	Blue light (3 W)	H_2_O	3	92
4	Blue light (7 W)	H_2_O	3	97
5	Blue light (5 W)	–	8	47
6	Blue light (5 W)	MeOH	3	61
7	Blue light (5 W)	DCM	20	18
8	Blue light (5 W)	EtOAc	3	65
9	Blue light (5 W)	CH_3_CN	3	72
10	Blue light (5 W)	DMSO	20	28
11	Blue light (5 W)	THF	15	19
12	Blue light (5 W)	EtOH	3	73
13	Blue light (5 W)	toluene	15	27
14	White light (5 W)	H_2_O	3	88
15	Green light (5 W)	H_2_O	3	91

^a^Reaction conditions: barbituric acid, benzaldehyde, and malononitrile were incorporated with 3DPAFIPN in stoichiometric amounts of 1 mmol each and a photocatalyst quantity of 0.2 mol%.

**Table 3:**
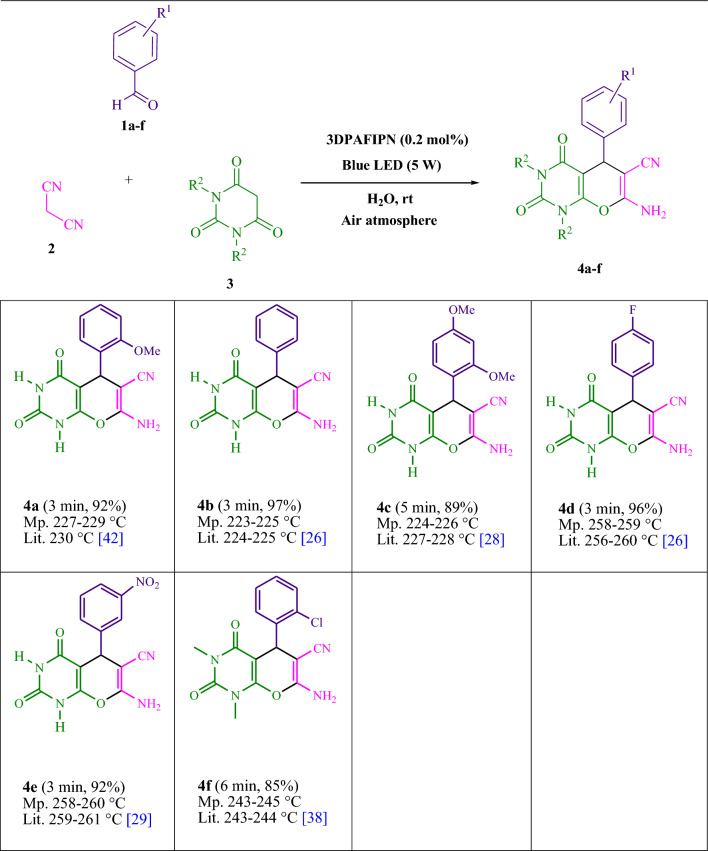
The production of pyrano[2,3-*d*]pyrimidine scaffolds is achieved through the implementation of the halogenated dicyanobenzene-based photosensitizer; 3DPAFIPN.

**Table 4:** The objective is to ascertain the values of turnover number (TON) and turnover frequency (TOF).

Entry	Product	TON	TOF
1	**4a**	460	153.3
2	**4b**	485	161.6
3	**4c**	445	89
4	**4d**	480	160
5	**4e**	460	153.3
6	**4f**	425	70.8

### Control experiments

**Figure 3:**
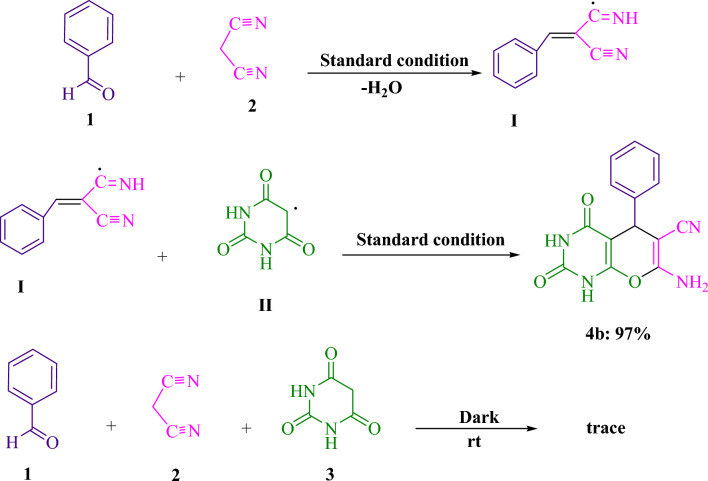
The investigation of the mechanisms underlying the reactions that feature barbituric acid (**3**, 1.0 mmol), malononitrile (**2**, 1.0 mmol), and benzaldehyde (**1**, 1.0 mmol) is facilitated by the performance of relevant control tests.

**Figure 4:**
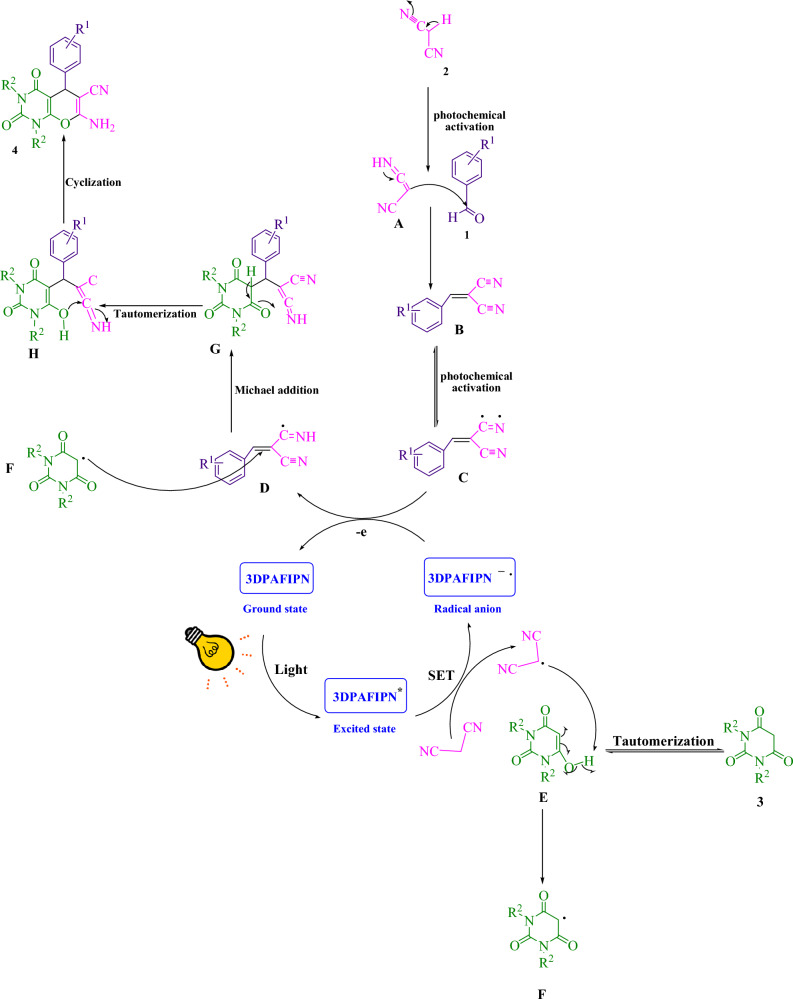
A comprehensive elucidation of the synthetic procedure for the generation of pyrano[2,3-*d*]pyrimidine frameworks is proffered herein.

Figure 3 illustrates the findings of the control experiments executed in order to elucidate the mechanism underlying the tripartite visible light-induced reaction. The initial step in the Knoevenagel–Michael cyclocondensation reaction involves the formation of arylidenemalononitrile (**I**), while the subsequent step entails its combination with (**II**). Under conventional conditions, the condensation of benzaldehyde (**1**) and malononitrile (**2**) was carried out with reduced water content, utilizing 3DPAFIPN in H_2_O under blue LED illumination. This resulted in the formation of arylidenemalononitrile (**I**). Subsequent to conducting experiments, it has been established that the interaction between arylidenemalononitrile (**I**) and barbituric acid radical (**II**) afforded the intended product, **4b**, at an impressive yield of 97%, following standard procedure. Product **4b** was found to exhibit detectable levels of production even amidst the absence of light during the reaction. As per the outcomes derived from this experiment, Fig. 4 presents a coherent and rational chemical course.

### The suggested mechanism

Figure 4 presents a comprehensive depiction of the suggested methodology. Through the implementation of single electron transfer (SET) mechanisms, the cyanoarene organic dye 3DPAFIPN has been utilized to develop photocatalytic tools that harness visible light energy as a sustainable resource. The use of visible light hastens the process. The malononitrile radical is generated through a strategy stemming from the single electron transfer (SET) mechanism, which augments 3DPAFIPN^*^ and is activated by visible light. The radical adduct (**C**) and the radical anion of 3DPAFIPN undergo electron transfer (ET), leading to the formation of intermediates (**D**) as well as the ground state 3DPAFIPN. The intermediate (**F**) is formed via the hydrogen atom abstraction of intermediate (**E**) by the radical of malononitrile. The intermediates denoted as (**F**) and (**D**) undergo a Michael acceptor reaction, leading to the formation of (**G**). Subsequently, (**4**) is generated through intramolecular cyclization and tautomerization processes.

Table 5 presents a comparative analysis of the efficacy of various catalysts in facilitating the formation of pyrano[2,3-*d*]pyrimidine frameworks. The approach in question employs minute quantities of photocatalysts and precipitates prompt chemical transformations whilst abstaining from the production of residual substances. This modality can be utilized in circumstances that involve observable wavelengths of light. Atom-economical protocols exhibit pronounced potency and exert substantial influence on the industrial domain at multigram levels.

**Table 5:** This study aims to investigate the catalytic activity of different catalysts utilized for the synthesis of **4b**^a^.

Entry	Catalyst	Conditions	Time/yield (%)	References
1	[DABCO](SO_3_H)_2_(Cl)_2_	H_2_O, Reflux	10 min/86	^26^
2	[DABCO](SO_3_H)_2_(HSO_2_)_2_	H_2_O, 90 °C	7 min/90	^26^
3	iron ore pellet	EtOH/H_2_O, Reflux	8 min/73	^28^
4	nano-sawdust-OSO_3_H	EtOH, Reflux	15 min/94	^29^
5	Al-HMS-20	EtOH, rt	12 h/92	^30^
6	TSA	EtOH/H_2_O, Reflux	90 min/88	^31^
7	B(OH)_3_	THF/H_2_O, Reflux	125 min/81	^31^
8	cellulose-based nanocomposite	THF/H_2_O, Reflux	35 min/90	^33^
9	DBA	EtOH/H_2_O, Reflux	58 min/94	^34^
10	3DPAFIPN	Blue LED, H_2_O, rt	3 min/97	This work

^a^The three-component synthesis employs benzaldehyde, malononitrile, and barbituric acid.

## Conclusion

The Knoevenagel–Michael cyclocondensation reaction, a radical-induced process, has been utilized to green photosynthesize pyrano[2,3-*d*]pyrimidine scaffolds from a combination of aryl aldehydes, malononitrile, and either barbituric acid or 1,3-dimethylbarbituric acid. The present study utilized the novel halogenated dicyanobenzene-based photosensitizer; 3DPAFIPN as a donor–acceptor (D–A) photocatalyst which operates via a consecutive visible-light-induced electron transfer process. Under room temperature and in an air environment, the utilization of blue light emitting diode (LED) technology has been demonstrated to yield a sustainable energy generation mechanism within an aqueous medium. The presented method offers various advantages to the field of chemical synthesis. These benefits encompass a swift response time, nullification of perilous solvents, augmented product yields, streamlined reaction mechanism, durable conditions, and employment of a sustainable energy resource. The utilization of chromatography was not deemed necessary for the separation protocol. Through the preservation of the end result, it is plausible to accelerate a multigram-scale reaction of exemplar substrates. Thus, the approach can be implemented within a context that upholds long-term ecological and financial viability.

### Supplementary Information

Supplementary Information.

## Data Availability

All data generated or analyzed during this study are included in this published article [and its supplementary information files].
