# Supplementary material for: 3DPAFIPN as a halogenated dicyanobenzene-based photosensitizer catalyzed gram-scale photosynthesis of pyrano[2,3-d]pyrimidine scaffolds
Source: Sci Rep. 2023 Aug 12;13:13142. doi: 10.1038/s41598-023-40360-w (PMC10423215; doi:10.1038/s41598-023-40360-w)
Supplement: Supplementary file 1 — Supplementary Information. [file 41598_2023_40360_MOESM1_ESM.pdf]

Supporting Information

3DPAFIPN as a halogenated dicyanobenzene-based photosensitizer catalyzed gram-scale photosynthesis of pyrano[2,3-*d*]pyrimidine scaffolds

Farzaneh Mohamadpour [*]

School of Engineering, Apadana Institute of Higher Education, Shiraz, Iran

* *Corresponding author. mohamadpour.f.7@gmail.com*

Contents

1. **Control experiments**

1.1. Control experiments on the 4b synthesis

1.2. Control experiments on intermediate A

1.3. The reaction mechanism's response to daylight and darkness

1.4. The impact of reaction temperature

1.5. Time's impact on the yield reaction

1.6. Visible light's impact on the reaction

2. ^{1}HNMR data for compounds (4a, 4b, 4c, 4d, 4e, and 4f)

3. ^{1}HNMR files for compounds (4a, 4b, 4c, 4d, 4e, and 4f)

1. Control experiments

1. 1. Control experiments on the 4b synthesis

Between benzaldehyde, malononitrile, and barbituric acid, Figure S1 explain the many strategies that have been employed in order to ascertain the necessity of the visible light source and the photocatalyst to reach the activation phase. Experimental investigations utilizing intermediate substances without a photocatalyst were carried out as a control. When the steps of the process involved the reaction of barbituric acid, benzaldehyde, and malononitrile, it was found that **4b** of product was only produced in negligible amounts when carried out at room temperature or in H_2O reflux conditions without the presence of blue light/3DPAFIPN, as shown in Figure S1: I and II. In the reaction involving barbituric acid, benzaldehyde, and malononitrile, as shown in Figure S1: III, 21% of **4b** of the chemical was generated when exposed to blue light, H_2O, and at room temperature without 3DPAFIPN. A trace amount of **4b** of product was also formed in the reaction of barbituric acid, benzaldehyde, and malononitrile in H_2O solution employing the photocatalyst; 3DPAFIPN without the use of blue light (Figure S1: IV).

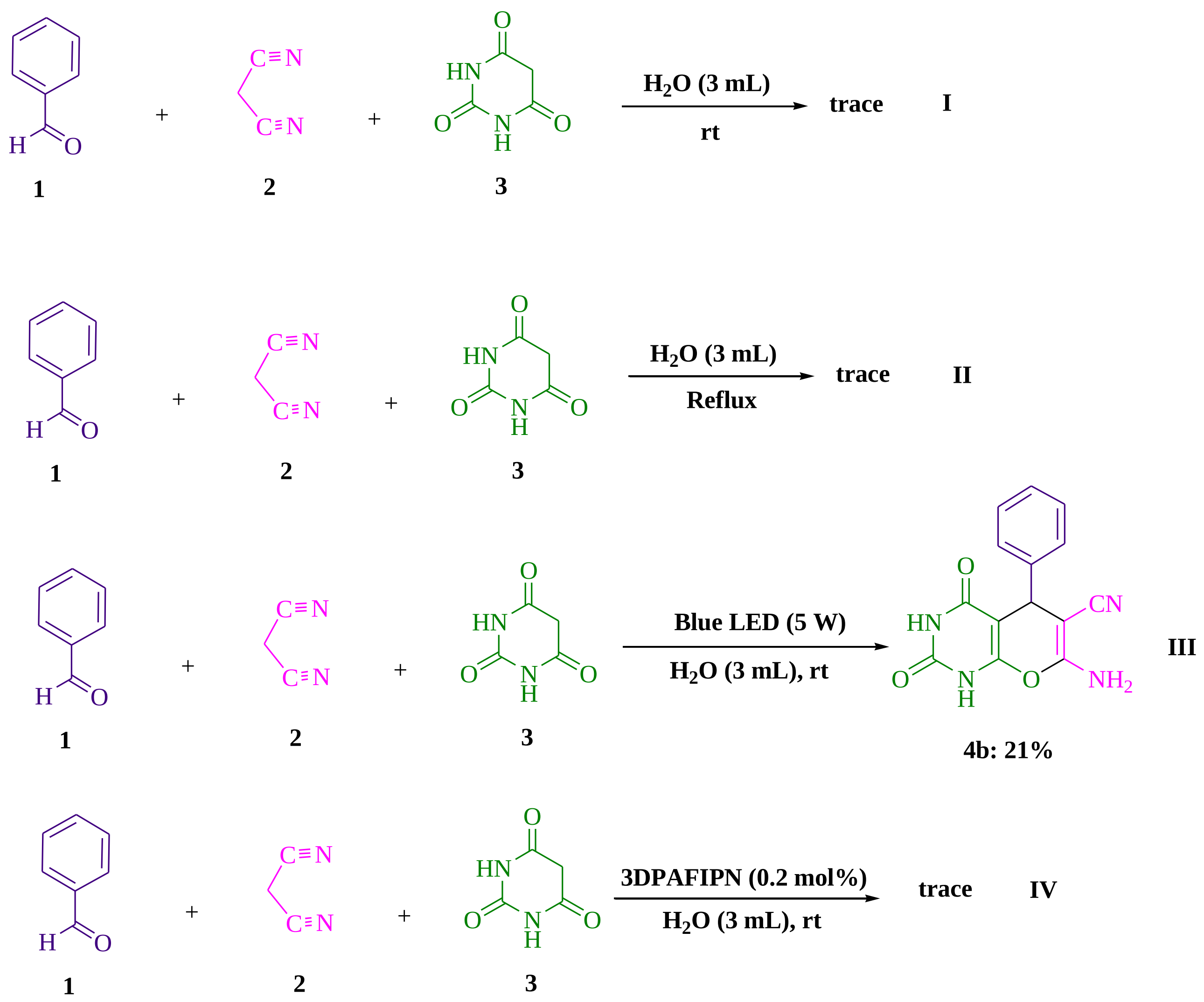

Figure S1. Control experiments on the **4b** synthesis.

1. 2. Control experiments on intermediate A

Experiments with the intermediate were conducted to offer additional experimental data for control objectives. The Knoevenagel-Michael cyclic condensation reaction's mechanism can be characterized as a procedure involving the successive occurrence of two different steps. Arylidenemalononitrile (**A**) is created in the first step, and then the product is condensed with barbituric acid (**3**) in the second. Using a 3DPAFIPN and H_2O solution, benzaldehyde (**1b**) and malononitrile (**2**) were agitated by exposure to visible light. The intermediate (**A**) was found in 92% of cases, as shown in Figure S2: I. Additionally, a tiny quantity of **4b** of product was produced when (**A**) was stirred at room temperature or refluxed in an H_2O solution containing barbituric acid (**3**) without the photocatalyst and visible light (as seen in Figure S2: II, III). In contrast, a small quantity of compound **4b** was produced when compound (**A**) and barbituric acid (**3**) were combined with 3DPAFIPN and H_2O in the absence of visible light (as shown in Figure S2: IV). In addition, a minuscule amount of **4b** was created when compound (**A**) and barbituric acid (**3**) were combined with blue light and H_2O in the absence of the 3DPAFIPN photocatalyst (as seen in Figure S2: V). The outcomes of the controlled studies showed that 3DPAFIPN and blue LED exposure were both necessary for the advancement of the reactions under consideration.

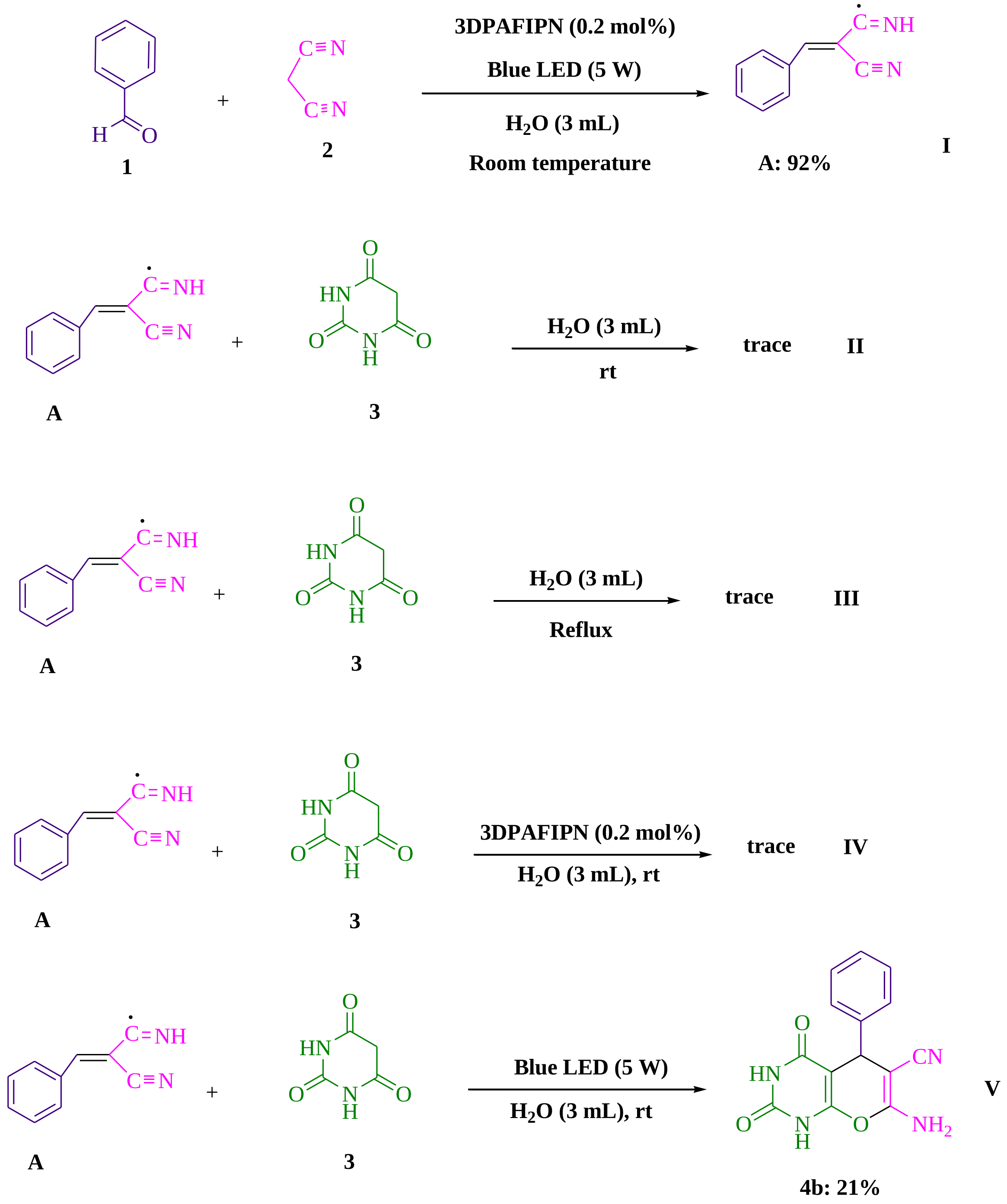

Figure S2. Control experiments on intermediate **A**.

1. 3. The reaction mechanism's response to daylight and darkness

The primary goal of the current work is to better understand how visible light and darkness/daylight interact with one another in chemical reaction mechanisms. This investigation examines a number of reactions to assess how darkness and daylight affect the efficacy of H_2O (3 mL) and 3DPAFIPN (0.2 mol%). Observations indicate that the emission of this chemical is significantly reduced when observable light wavelengths are disregarded.

The yield (%) obtained for the **4b** synthesis under the influence of daylight: trace

The yield (%) obtained for the **4b** synthesis under the influence of darkness: trace

1. 4. The impact of reaction temperature

To confirm the necessity of visible light radiation for the phase under test, a number of control experiments were carried out using tried and true techniques. To determine the effects of exposure to visible light radiation on the mentioned process, a research investigation was carried out. It has been demonstrated that these reactions can only be brought on by prolonged exposure to light. In the absence of visible light irradiation, low product yields and delayed reaction kinetics were seen. The fact of the matter is that reactions are started by using radiation from visible light sources. As illustrated in Figure S3, blue LEDs, also known as light-emitting diodes, serve as a significant source of visible light energy that is widely applied in many processes.

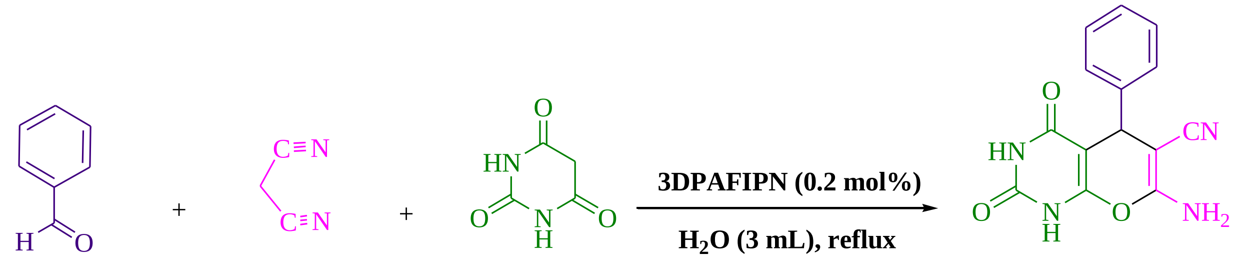

Figure S3. The impact of reaction temperature.

1. 5. Time's impact on the yield reaction

The inquiry also revealed that the presence of 3DPAFIPN, blue light irradiation, and H_2O is necessary to induce the outcomes, confirming them as important factors in determining the successful conclusion of the "investigation". Additionally, it was found that, as shown by the data in Table S1, an increase in reaction time had no discernible impact on the output reaction yield.

Table S1. A time-based optimization table for **4b** production[a]

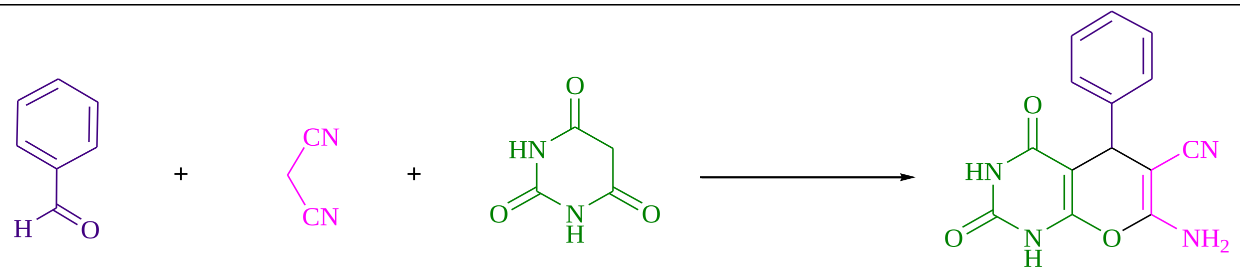

Entry	Photocatalyst	Solvent (3 mL)	Time (min)	Isolated Yields (%)
1	3DPAFIPN (0.2 mol%)	H_2O	10	97
2	3DPAFIPN (0.2 mol%)	H_2O	20	97

[a]Reaction conditions: the mixture of barbituric acid (1.0 mmol), malononitrile (1.0 mmol), and benzaldehyde (1.0 mmol) is used in the test technique, which involves illuminating the sample with a 5 W blue LED at room temperature.

1. **6. Visible light's impact on the reaction**

According to recent studies, a visible light source must be used to achieve meaningful conversion rates. It has been demonstrated that the procedure cannot be adequately carried out by conventional heating to temperatures of 90–100 °C under normal air conditions. Rapid chemical transformations result from the fast oscillating motion of the chemical bonds, which are made possible by the luminescence of visible light, and the frequent and quick encounters of the reactants. The findings of the experiment demonstrate, 3DPAFIPN undergoes a quick transition

from the ground state to the excited state via the contact system. The simultaneous impacts of exposure to visible light and 3DPAFIPN may be to blame for the accelerated chemical reaction rate. A viable replacement for transition metal-based redox photocatalysts has been shown for organic photocatalysts, such as 3DPAFIPN, because of their favorable economic and environmental characteristics. The photochemical behavior of 3DPAFIPN in response to stimulation by visible light was thoroughly studied. These investigations showed that 3DPAFIPN exhibits a quick inter-system transition to the triplet state with the lowest energy. Due to its inherent synthesis efficiency and potential, 3DPAFIPN demonstrates amazing light-absorbing qualities and has a lot of potential for use in a variety of visible light-catalyzed chemical processes.

Regardless of the concurrent usage of 3DPAFIPN, Table 2, Item 2 documents the execution of the chemical reaction in low light. The outcomes of controlled studies demonstrate that, whether or not there is daylight present, the reaction efficiency is minimal in the absence of visible light. The **4b** product matches the exact molecule listed as item 2 in Table 2 according to the experimental findings of the current study. Additionally, 3DPAFIPN's extraordinary redox potential range, extended excited state time, and improved fluorescence quantum yield are likely to contribute to the benefits of its catalytic capacity. The 3DPAFIPN photocatalyst is incredibly effective in accelerating several organic transformations. The current photocatalyst has a rather low efficiency as a unipolar electron acceptor and donor in the unexcited ground state. However,

the aforementioned material achieves an excited state upon exposure to visible light, which functions as a powerful catalyst for single electron transfer.

2. ^{1}HNMR data for compounds (4a, 4b, 4c, 4d, 4e, and 4f)

7-Amino-5-(2-methoxyphenyl)-2,4-dioxo-2,3,4,5-tetrahydro-1H-pyrano[2,3-d]-pyrimidine-6-carbonitrile (4a)

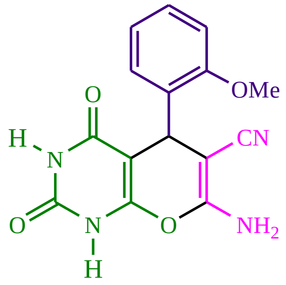

Yield: 92%; m.p. 227-229 °C; ^{1}HNMR (300 MHz, DMSO-d_6): 3.79 (3H, s, OCH_3), 4.49 (1H, s, CHAr), 7.46-7.78 (4H, m, ArH & NH_2), 8.18 (2H, t, J = 8.0 Hz, ArH), 10.58 (1H, s, NH), 11.36 (1H, s, NH).

7-amino-2,4-dioxo-5-phenyl-2,3,4,5-tetrahydro-1H-pyrano[2,3-d]pyrimidine-6-carbonitrile (4b)

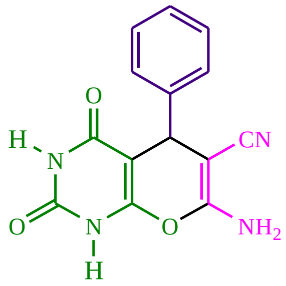

Yield: 97%; m.p. 223-225 °C; ^{1}HNMR (400 MHz, DMSO-d_6): 4.24 (1H, s, CHAr), 7.90-8.09 (7H, m, ArH & NH_2), 10.65 (1H, s, NH), 11.82 (1H, s, NH).

7-Amino-5-(2,4-dimethoxyphenyl)-2,4-dioxo-2,3,4,5-tetrahydro-1H-pyrano[2,3-d]-pyrimidine-6-carbonitrile (4c)

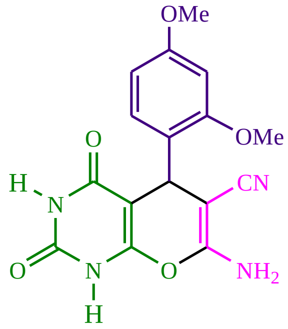

Yield: 89%; m.p. 224-226 °C; ^{1}HNMR (300 MHz, DMSO-d_6): 3.71 (3H, s, OCH_3), 3.76 (3H, s, OCH_3), 4.56 (1H, s, CHAr), 7.13-7.53 (5H, m, ArH & NH_2), 10.04 (1H, s, NH), 11.29 (1H, s, NH).

7-Amino-5-(4-fluorophenyl)-2,4-dioxo-2,3,4,5-tetrahydro-1H-pyrano[2,3-d]-pyrimidine-6-carbonitrile (4d)

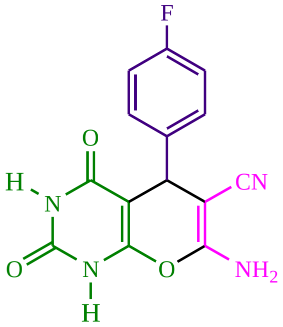

Yield: 96%; m.p. 258-259 °C; ^{1}HNMR (300 MHz, DMSO-d_6): 4.28 (1H, s, CHAr), 7.18-8.62 (6H, m, ArH & NH_2), 10.73 (1H, s, NH), 11.83 (1H, s, NH).

7-amino-5-(3-nitrophenyl)-2,4-dioxo-2,3,4,5-tetrahydro-1H-pyrano[2,3-d]pyrimidine-6-carbonitrile (4e)

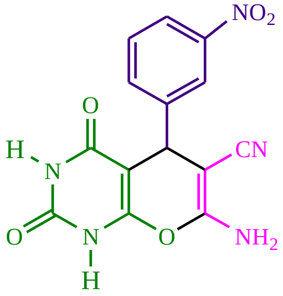

Yield: 92%; m.p. 258-260 °C; ^{1}HNMR (400 MHz, DMSO-d_6): 4.21 (1H, s, CHAr), 7.82-8.19 (6H, m, ArH & NH_2), 10.53 (1H, s, NH), 11.77 (1H, s, NH).

7-Amino-5-(2-chlorophenyl)-1,3-dimethyl-2,4-dioxo-2,3,4,5-tetrahydro-1H-pyrano[2,3-d]-pyrimidine-6-carbonitrile (4f)

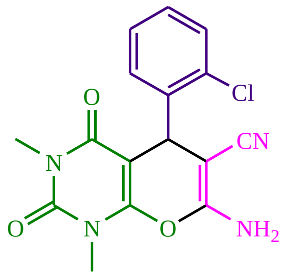

Yield: 85%; m.p. 243-245 °C; ^{1}HNMR (400 MHz, DMSO-d_6): 3.31 (3H, s, NCH_3), 3.36 (3H, s, NCH_3), 4.51 (1H, s, CHAr), 7.53-7.59 (4H, m, ArH & NH_2), 8.18 (2H, t, J = 6.8 Hz, ArH).

3. ^{1}HNMR files for compounds (4a, 4b, 4c, 4d, 4e, and 4f)

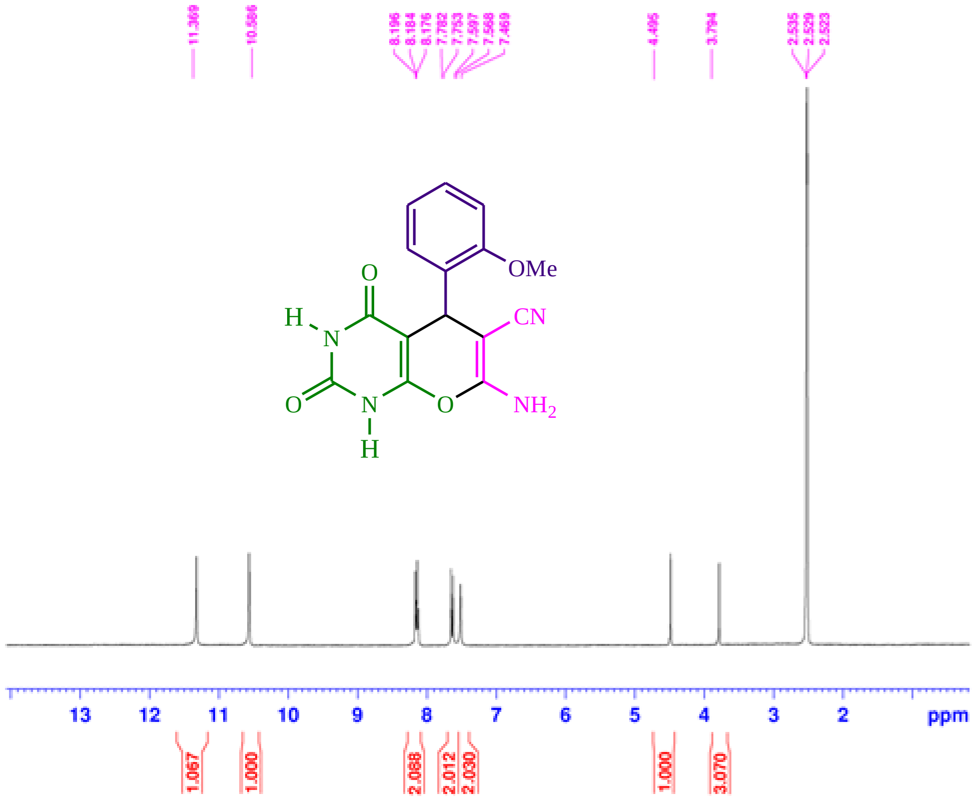

Figure S4. ^{1}HNMR Spectrum of compound (300 MHz, DMSO-d_6) of **4a**

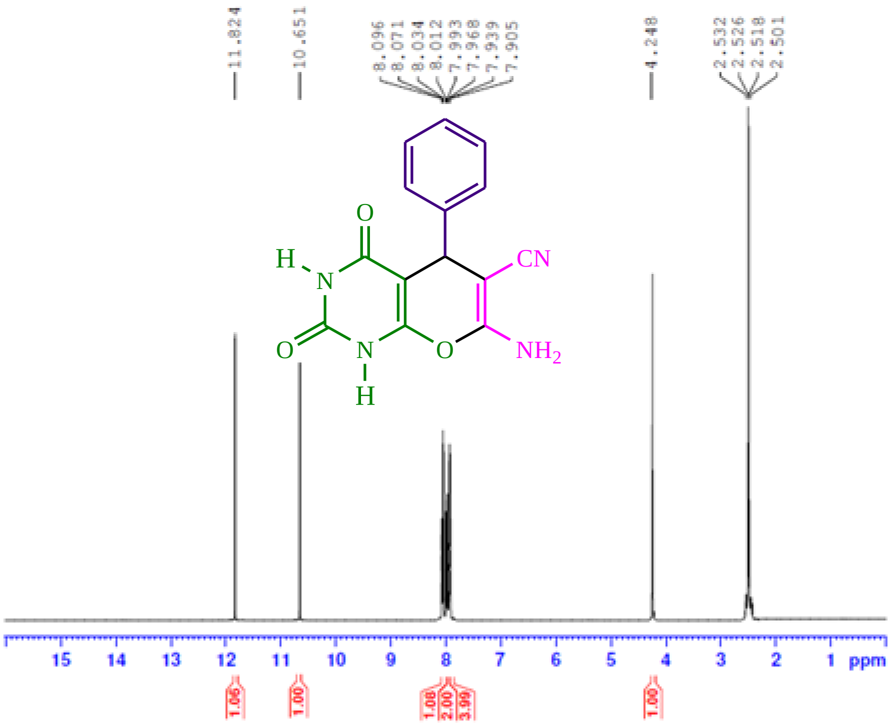

Figure S5. ^{1}HNMR Spectrum of compound (400 MHz, DMSO-d_6) of **4b**

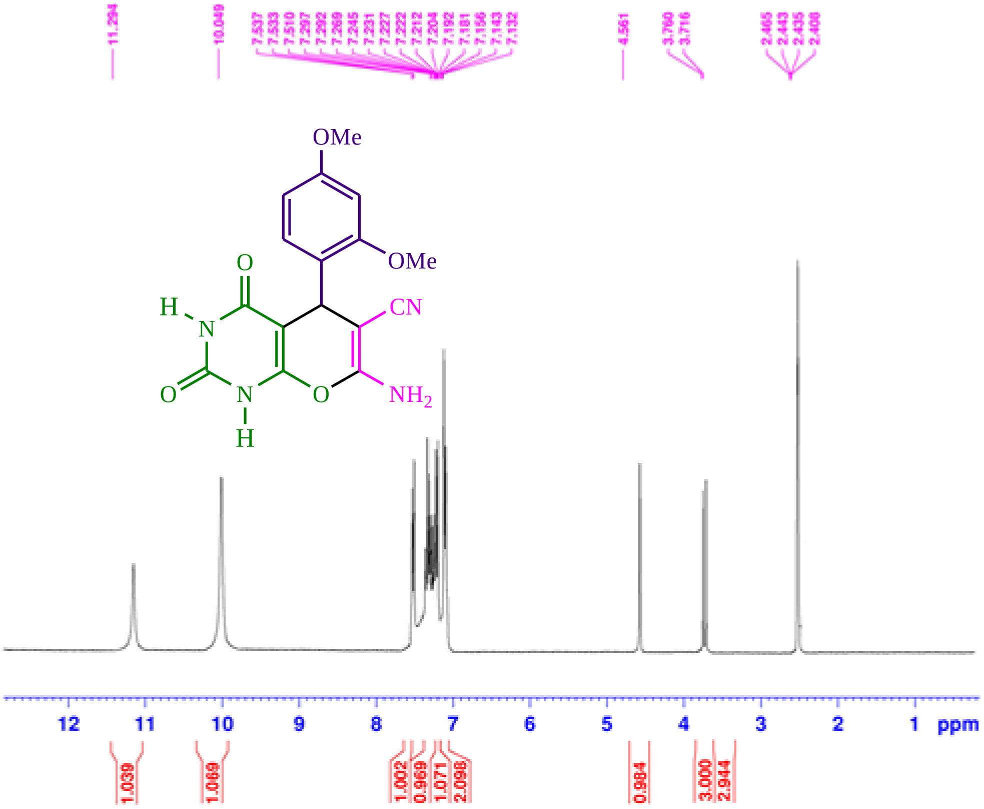

Figure S6. ^{1}HNMR Spectrum of compound (300 MHz, DMSO-d_6) of **4c**

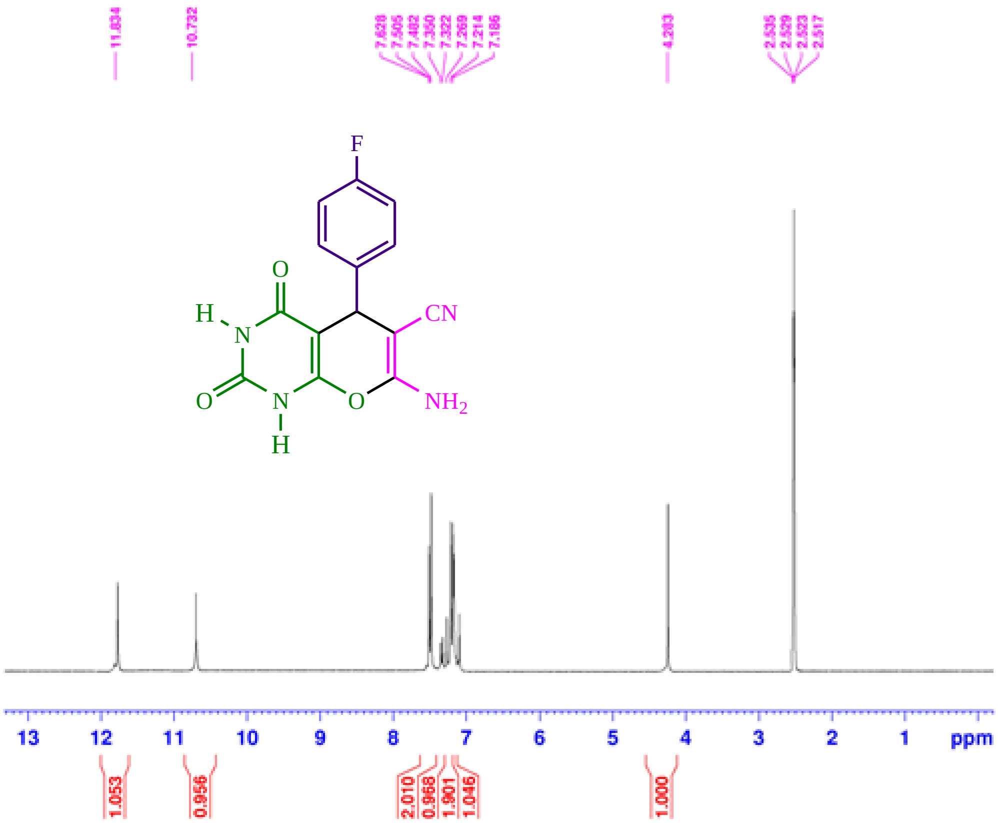

Figure S7. ^{1}HNMR Spectrum of compound (300 MHz, DMSO-d_6) of **4d**

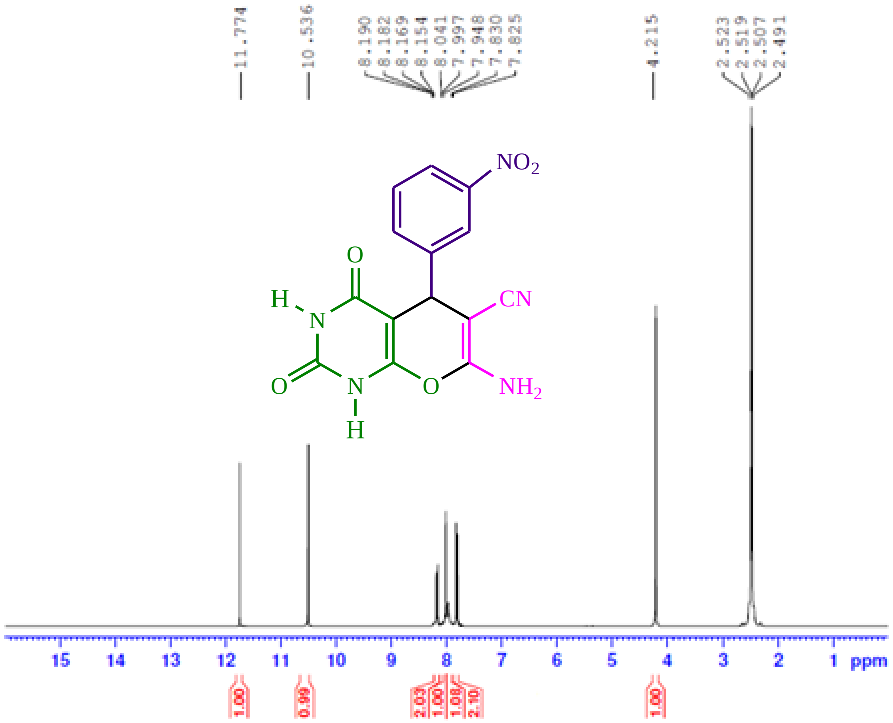

Figure S8.^{1}HNMR Spectrum of compound (400 MHz, DMSO-d$_6$) of **4e**

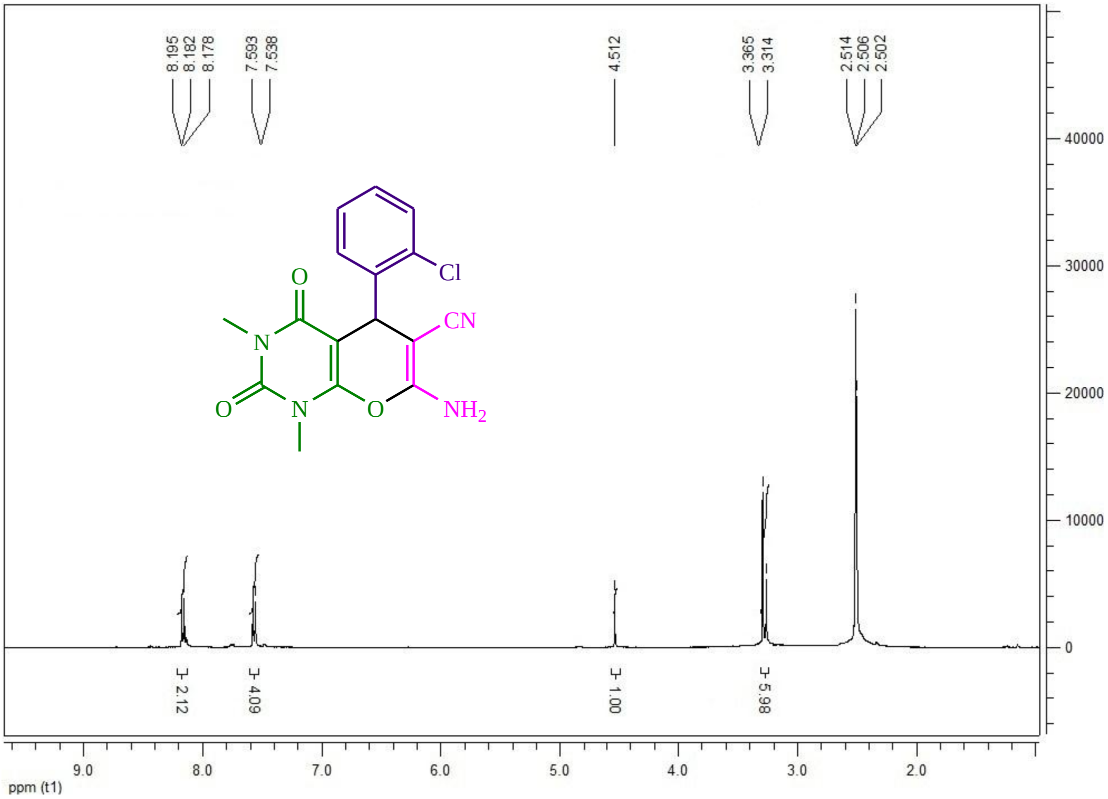

Figure S9. ^{1}HNMR Spectrum of compound (400 MHz, DMSO-d$_6$) of **4f**
